# Synchronous Human Papillomavirus (HPV)-Positive Oropharyngeal Carcinoma and Occult Papillary Thyroid Carcinoma: A Case Report and Review of the Literature

**DOI:** 10.7759/cureus.89385

**Published:** 2025-08-04

**Authors:** Tung-Sen Tai, Yun-An Chen, Chun-Wei Huang

**Affiliations:** 1 Department of Education, China Medical University Hospital, Taichung, TWN; 2 Department of Pathology, China Medical University Hospital, Taichung, TWN; 3 Department of Otolaryngology – Head and Neck Surgery, China Medical University Hospital, Taichung, TWN

**Keywords:** hpv-positive oropharyngeal squamous cell carcinoma, induction chemotherapy, level ii cervical lymph node metastasis, occult thyroid carcinoma, papillary thyroid carcinoma, synchronous malignancy, transoral robotic surgery

## Abstract

Synchronous malignancies involving the oropharynx and thyroid gland are rare. We report the case of a 52-year-old female diagnosed with human papillomavirus (HPV)-associated oropharyngeal squamous cell carcinoma (OPSCC) with a concurrent, clinically occult papillary thyroid carcinoma (PTC). The patient initially presented with HPV-associated OPSCC and concerns for cervical lymphadenopathy, presumed to be linked to regional metastasis from the oropharyngeal primary. However, further pathological evaluation revealed that one of the metastatic lymph nodes within the same cervical level originated from an undiagnosed PTC. This report highlights the need for careful pathological evaluation and consideration of multiple primary tumors in patients presenting with cervical lymph node metastases.

## Introduction

Synchronous primary malignancies, defined as two or more distinct primary tumors diagnosed simultaneously or within six months of each other, occur in the head and neck region and have an estimated incidence of 2-4% per year [1]. These malignancies, including oropharyngeal squamous cell carcinoma (OPSCC), frequently share etiological factors such as tobacco use and alcohol consumption [1]. In recent years, however, there has been a marked increase in the incidence of human papillomavirus (HPV)-related OPSCC, particularly affecting the tonsillar region. HPV-positive OPSCC represents a distinct clinical and molecular entity, demonstrating enhanced responsiveness to treatment and a significantly more favorable prognosis compared to HPV-negative disease [2]. Papillary thyroid carcinoma (PTC), the most common endocrine malignancy, typically follows an indolent course and may remain asymptomatic, often not requiring immediate intervention [3]. The development of thyroid carcinoma is influenced by multiple risk factors, including ionizing radiation exposure, positive family history, and the use of alcohol or tobacco [3].

In patients with HPV-related OPSCC, the presence of cervical lymphadenopathy is generally indicative of regional metastatic spread [4]. However, the presence of nodal metastases may obscure the coexistence of a second primary malignancy. This report presents a rare case of synchronous HPV-positive OPSCC and occult PTC, in which cervical lymph node metastases were initially presumed to originate solely from the oropharyngeal primary but were later found to include a separate lymph node harboring metastatic PTC within the same cervical region.

## Case presentation

**Figure 1:**
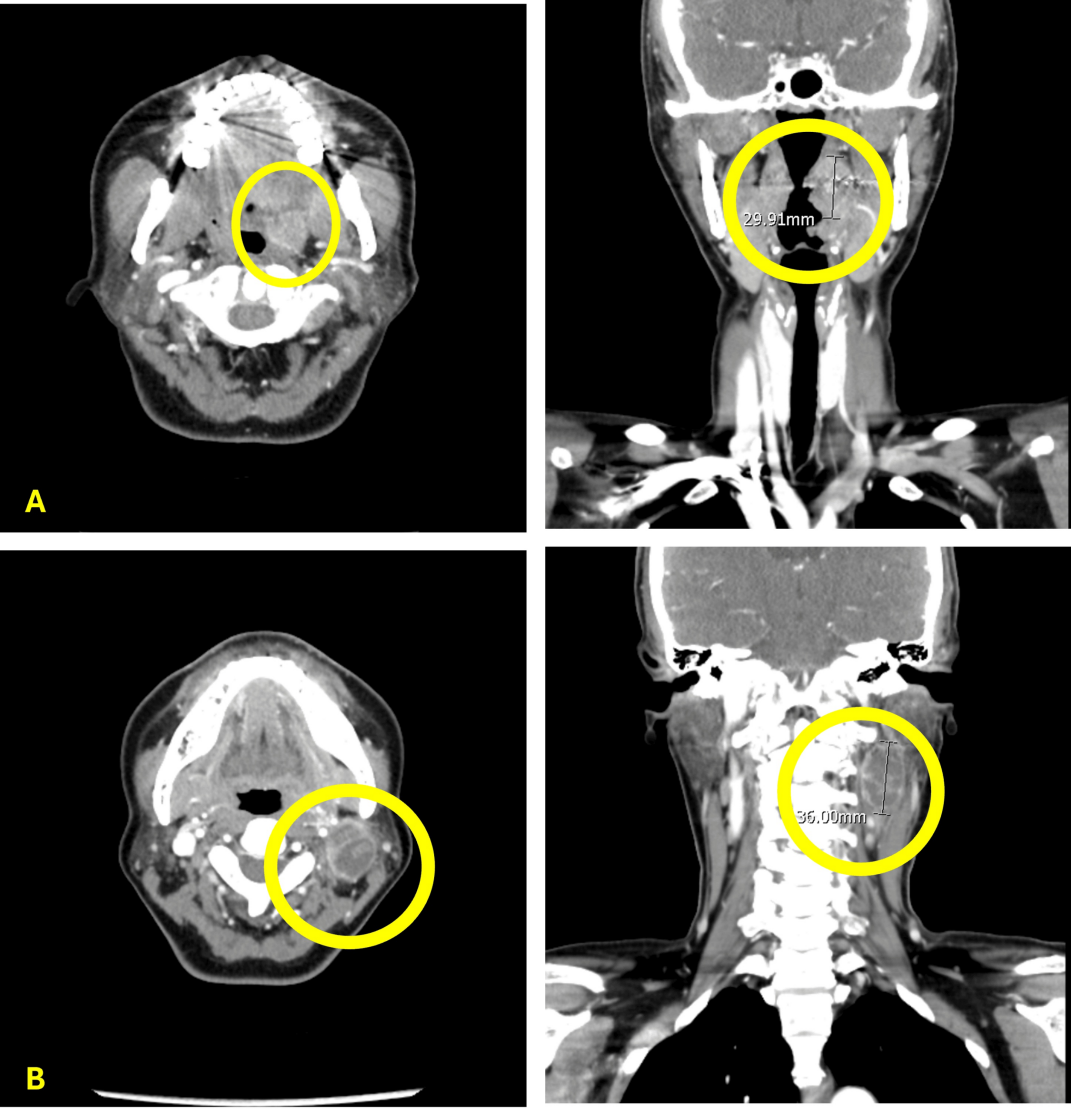
CT findings before the induction chemotherapy A.The images on the upper panel revealed an asymmetrically enlarged left tonsil, approximately 3 cm in its largest diameter, suspicious for tonsillar carcinoma. B. The images on the lower panel revealed a 3.6 cm cystic degenerative change at the right level II neck region, suspicious for lymph node metastasis CT: computed tomography

A 52-year-old female patient, with no known history of systemic disease, relevant family history, or social risk factors such as betel nut chewing, smoking, or alcohol consumption, presented to the otolaryngology outpatient department in September 2024 with a two-month history of pain and swelling in the left infra-auricular region. The swelling was initially suspected to be an infectious mass, and the patient was prescribed a course of antibiotics. However, the mass was refractory to antibiotic treatment, prompting further evaluation. Physical examination revealed an indurated tumor over the left tonsillar fossa. A punch biopsy of the left tonsillar tumor was subsequently performed, and histopathological analysis revealed non-keratinizing squamous cell carcinoma (SCC), with positive p16 immunohistochemical staining (a surrogate marker of HPV-associated malignancy). A CT scan of the head and neck was performed on September 27, 2024, for further assessment, which demonstrated a contrast-enhanced lesion measuring approximately 3 cm (the largest diameter) in the left tonsil, leading to the diagnosis of oropharyngeal cancer (Figure 1A). Additionally, an enlarged lymph node with cystic degeneration was identified in the left level II cervical region (Figure 1B).

**Figure 2:**
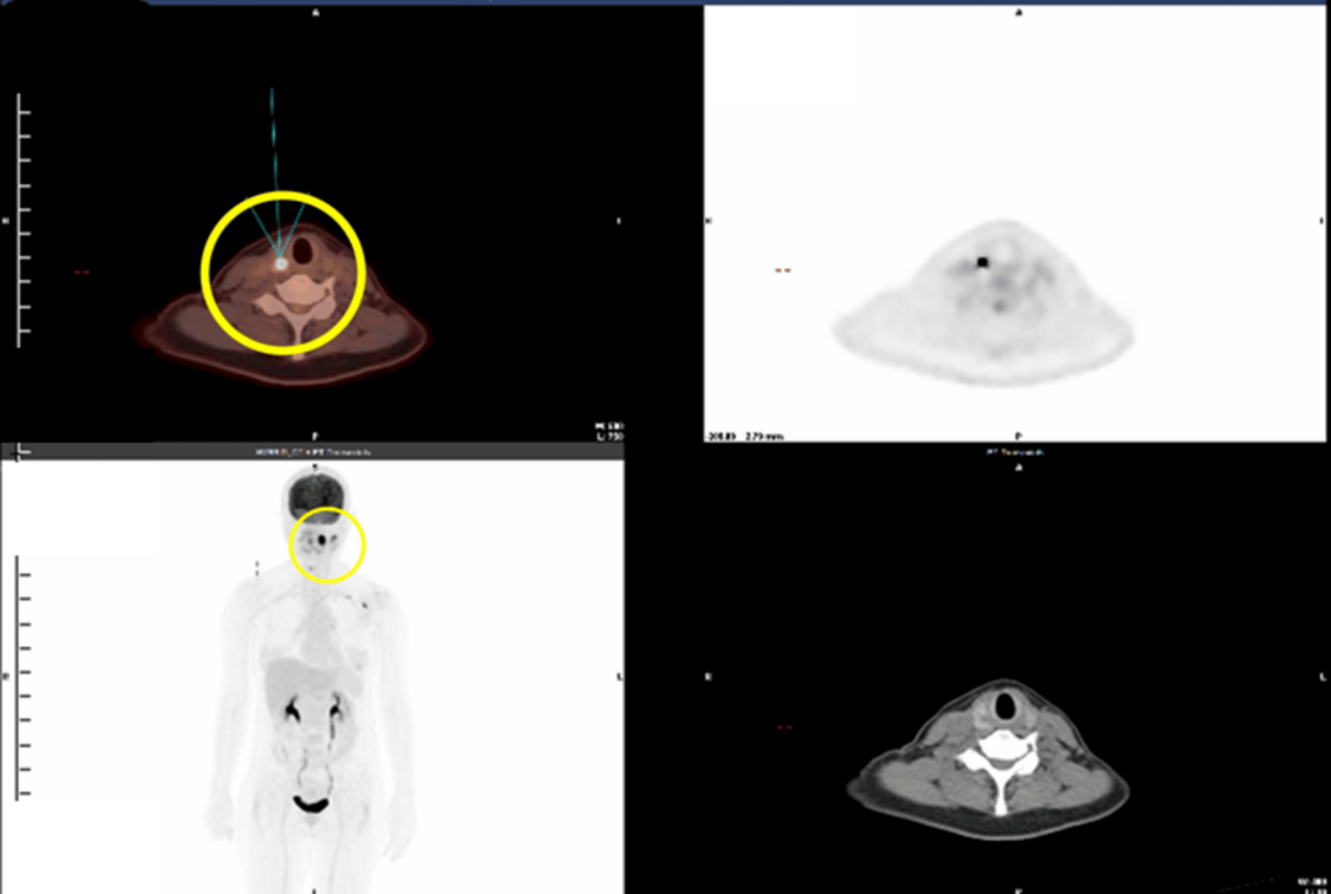
PET/CT showing an abnormal hypermetabolic nodule (about 1 cm, SUV max: 50.89) in the right thyroid lobe PET/CT: positron emission tomography/computed tomography; SUV max: maximum standardized uptake value

A PET/CT scan on October 11, 2024, revealed an abnormal hypermetabolic lesion (approximately 2.5 centimeters, maximum standardized uptake value (SUV max): 17.15) involving the left tonsil, consistent with a malignant tumor. An abnormal hypermetabolic lymph node with central necrosis (approximately 3.6 cm, SUV max: 10.50) was identified in the left neck level II, suggestive of regional metastasis. Furthermore, the PET/CT incidentally identified an abnormal hypermetabolic nodular lesion (approximately 1 cm, SUV max: 50.89) in the right lobe of the thyroid, which was suspected to be a synchronous primary malignant or metastatic tumor of the thyroid gland (Figure 2). The impression from the PET/CT scan was of a malignant tumor involving the left tonsil with at least one ipsilateral left neck lymph node metastasis and no distant metastasis. Based on imaging findings and the American Joint Committee on Cancer (AJCC) 8th edition staging criteria, the clinical stage of the oropharyngeal cancer was determined to be T2N1Mx [5]. Additionally, there was a highly suspicious lesion in the right thyroid lobe that should be proven.

After a thorough discussion regarding treatment options, the patient opted to undergo induction systemic therapy for the management of non-keratinizing SCC, p16 positive, classified as clinical T2N1 [5]. She received five cycles of induction chemotherapy in combination with targeted therapy with a regimen consisting of cetuximab, cisplatin, and docetaxel, beginning in October 2024. During the course of therapy, she was found to have chronic hepatitis B virus (HBV) infection. As chemotherapy can trigger HBV reactivation and lead to hepatic complications, antiviral prophylaxis with entecavir was initiated to prevent disease flare-up. During the fourth cycle of chemotherapy, the patient developed an upper respiratory tract infection, presenting with cough, sore throat, fever, and tachycardia. She was admitted to the hospital and treated with intravenous antibiotics, specifically cefepime. Due to febrile neutropenia and impaired liver function during the infection, the docetaxel dose was reduced during the fifth cycle of chemotherapy.

**Figure 3:**
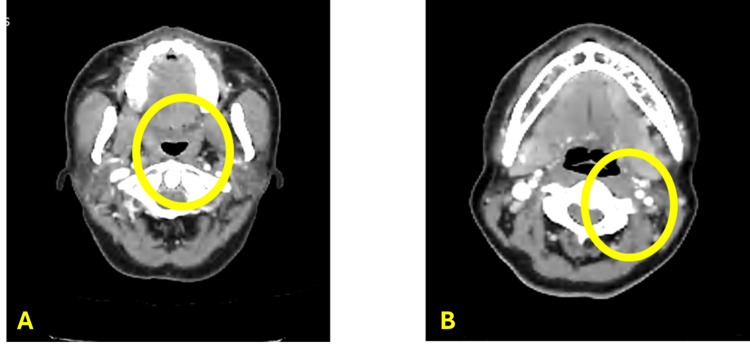
CT findings after the induction chemotherapy A.The image on the left side revealed complete interval shrinkage of the enhanced mass in the left palatine tonsil. B. The image on the right side also revealed complete shrinkage at the level II neck region CT: computed tomography

A second tumor assessment was conducted after five cycles of therapy, approximately four months after initiation of treatment. A follow-up CT scan of the head and neck was performed on December 26, 2024, to evaluate treatment response. According to the Response Evaluation Criteria in Solid Tumors (RECIST) version 1.1, a partial response is defined as the disappearance of all target lesions and a reduction in the short axis of any pathological lymph nodes to less than 10 mm [6]. The follow-up imaging demonstrated interval near-complete resolution of the enhancing mass in the left palatine tonsil (Figure 3A) and partial resolution of the previously enlarged lymph node in the left level II region (Figure 3B).

Neck ultrasonography was also performed on January 3, 2025, to further evaluate the thyroid lesion. The ultrasound revealed a 10.1 × 10.2 × 11.2 mm ill-defined, hypoechoic, and heterogeneous solid nodule in the right lobe of the thyroid. The thyroid lesion was suspected to represent a primary malignancy, and fine-needle aspiration biopsy was recommended. The cytological evaluation reported findings of atypia of undetermined significance (AUS), corresponding to category III of the Bethesda System for Reporting Thyroid Cytopathology [7]. After discussion with a hematology specialist and the patient, it was decided that the thyroid lesion would be addressed after definitive treatment for the oropharyngeal cancer. The patient subsequently underwent transoral robotic surgery (TORS) for the left tonsillar carcinoma and left modified radical neck dissection for residual disease on January 18, 2025.

**Figure 4:**
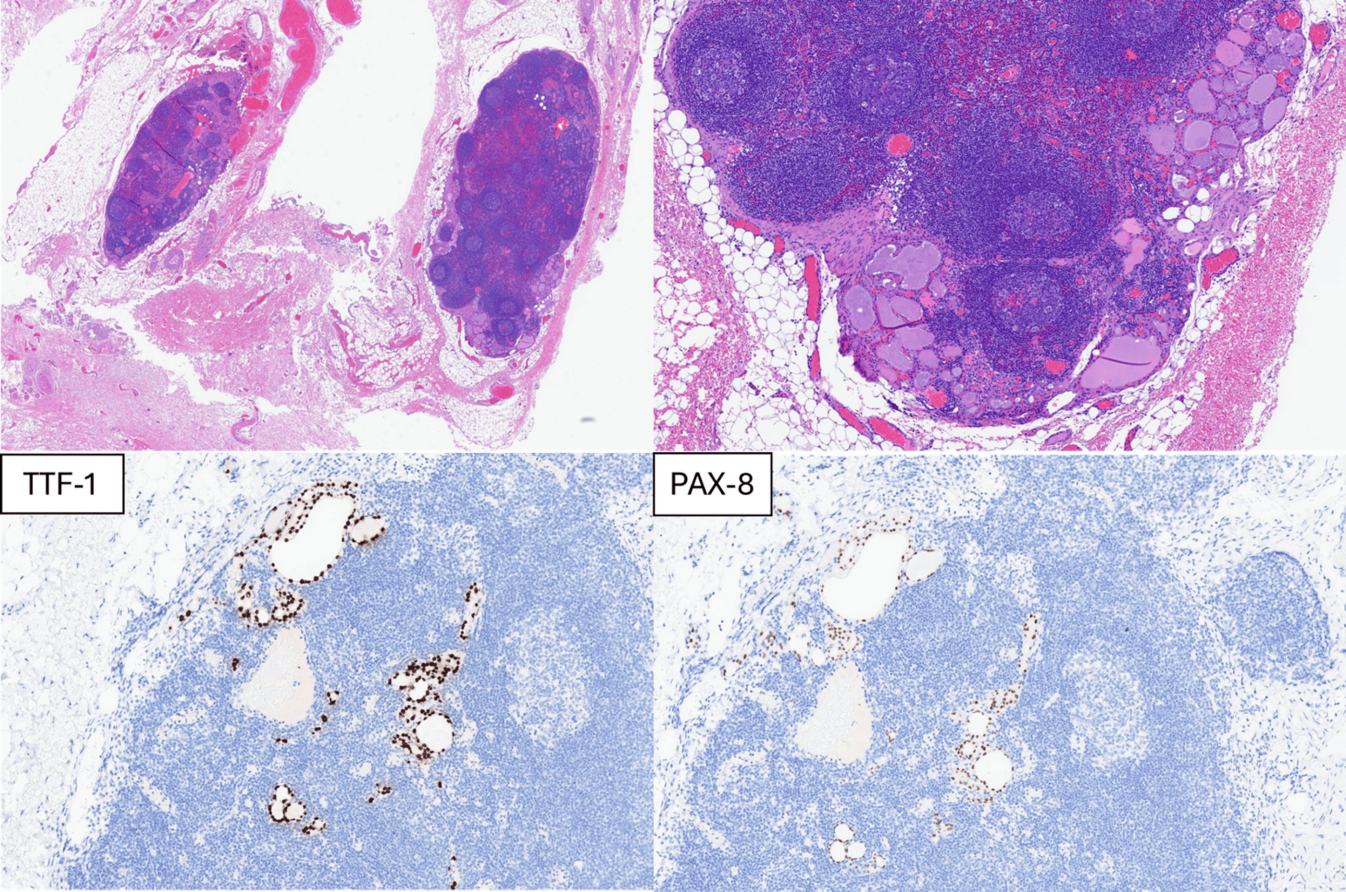
Histopathological and immunohistochemical findings suggestive of metastatic thyroid carcinoma in cervical lymph nodes The sections show lymph nodes with microscopic foci containing well-formed thyroid follicles and colloid production located in the subcapsular sinuses. The lining follicular cells exhibit mild cytological atypia; however, no definitive cytological features of conventional papillary thyroid carcinoma are identified. In these foci, no papillary structures or psammoma bodies are observed. However, these lymph nodes were found at neck levels II and III, which are not typical locations for ectopic thyroid tissue, as it is usually found at levels VI or VII. Therefore, metastasis from a thyroid carcinoma was highly suspected. Immunohistochemical stains, including TTF-1 (+) (lower left images) and PAX-8 (+) (lower right images), confirm the origin as thyroid follicular cells. A well-differentiated thyroid carcinoma of follicular cell origin was highly suspected

Pathological analysis revealed residual HPV-associated SCC of the left tonsil. Lymph nodes in the left neck levels I, IV, and V were negative for malignancy. However, in the left neck level II, one lymph node contained atypical thyroid tissue highly suspicious for metastatic differentiated thyroid carcinoma (DTC). Another lymph node in level III also contained atypical thyroid tissue, again highly suspicious for metastatic DTC (Figure 4). No residual SCC was identified in the lymphadenopathy. According to the AJCC 8th edition, the pathological stage group for oropharyngeal carcinoma was ypT1N0, clinical M0, corresponding to stage I [5].

**Figure 5:**
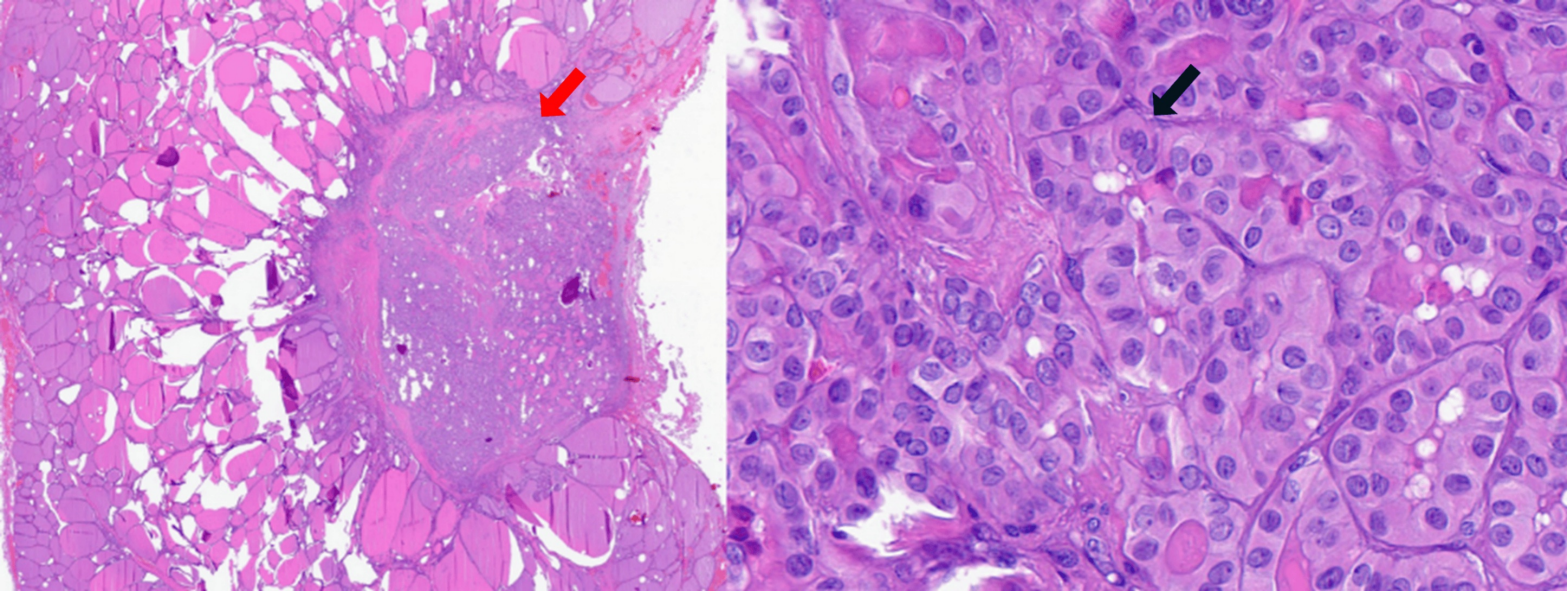
The figure demonstrates features consistent with classic papillary thyroid carcinoma The red arrow (left image) indicates a focus of malignancy within the thyroid tissue. The right panel demonstrates classic histopathological features of papillary thyroid carcinoma. Notably, the area highlighted by the black arrow exhibits characteristic nuclear features, including nuclear enlargement, elongation, and overlapping, which are hallmark findings in papillary thyroid carcinoma

Following a multidisciplinary team discussion, the patient was advised to undergo bilateral total thyroidectomy, followed by adjuvant radiotherapy (RT). One month after TORS, the patient was readmitted and underwent bilateral total thyroidectomy. Pathological examination identified a nodule measuring 0.7 × 0.5 × 0.4 cm in the right thyroid lobe. Histological evaluation revealed classic PTC (Figure 5). There was no evidence of tumor necrosis, angioinvasion, lymphatic invasion, perineural invasion, or extrathyroidal extension. According to the AJCC pathological tumor-node-metastasis (pTNM) classification and the previous neck dissection findings, the thyroid tumor was classified as pT1a (tumor ≤1 cm, confined to the thyroid) and pN1b (metastases to unilateral or contralateral lateral neck lymph nodes, levels II and III) [8]. As the patient was under 55 years of age, the final pathological stage was classified as stage I. Postoperative treatment plans included RT to the primary tumor site and cervical lymph node regions (levels I through III) with a total dose of 60 Gray (Gy), followed by radioactive iodine (RAI) therapy using iodine-131 (I-131).

The patient subsequently underwent adjuvant RT from March to April 2025. No significant adverse effects were reported during the treatment course, aside from mild neck tightness. Notably, there was no occurrence of dry mouth, sore throat, or oral ulceration. Follow-up laboratory testing revealed hypothyroidism, for which levothyroxine supplementation was initiated. A follow-up CT scan performed on May 23, 2025, demonstrated no evidence of recurrence in the oropharynx, thyroid gland, or cervical lymph nodes. The patient then received RAI therapy on July 3, 2025, with a total administered dose of 90 millicuries of iodine-131 sodium iodide. No post-treatment complications were observed. At the most recent follow-up, the patient remained clinically stable; she continues to undergo thyroid hormone replacement therapy and is under regular clinical surveillance.

## Discussion

Our patient, who had no identifiable social risk factors, was diagnosed with HPV-positive (p16-positive) OPSCC, clinically staged as T2N1aMx [5]. Treatment strategies for OPSCC include both surgical and non-surgical modalities. For patients clinically staged as T1 with a single involved lymph node measuring ≤3 cm (N1 stage), appropriate therapeutic options encompass single-modality RT, definitive concurrent chemoradiation, or surgical resection [9]. In terms of surgical management, recent advancements have favored minimally invasive techniques, notably TORS. TORS is particularly suitable for T1-T2 tumors arising within the tonsillar fossa due to the favorable potential for postoperative functional recovery, including speech, swallowing, and mastication. Consequently, these minimally invasive approaches have become increasingly important options. In our patient's scenario, the preservation of appearance and oral function was a significant consideration. After multidisciplinary consultation, induction chemotherapy before TORS was selected to initially reduce the tumor burden.

The induction chemotherapy regimen for our patient consisted of docetaxel combined with cisplatin and targeted therapy with cetuximab. Recent studies have shown that induction chemotherapy with taxane and platinum agents can effectively reduce the size of the primary tumor and regional lymph nodes in OPSCC. This reduction may allow for de-escalation of subsequent treatment and lead to partial or complete remission [10]. Furthermore, emerging research indicates that the addition of immunotherapy agents, such as nivolumab targeting the programmed death-ligand 1 (PD-L1) pathway, or target therapy agents, epidermal growth factor receptor (EGFR) inhibitors like cetuximab, with conventional cisplatin-based chemotherapy enhances tumor response rates without increasing overall treatment-related toxicity [10]. However, immunotherapy or targeted therapy as a standalone treatment has not yet demonstrated definitive benefit in the curative management of OPSCC [10].

Overall, these studies emphasize that concurrent platinum-based chemotherapy improves local tumor control and potentially eradicates micro-metastatic disease. Conversely, substituting cisplatin with cetuximab has been associated with inferior overall survival outcomes without a corresponding decrease in treatment-associated toxicities [11]. Our patient exhibited an excellent clinical response to induction chemotherapy, achieving near-complete remission. Follow-up CT imaging confirmed significant tumor remission, with the original 3-cm lesion nearly entirely resolved. Compared to conventional surgical approaches, TORS has demonstrated superior outcomes by facilitating precise tumor excision while minimizing functional impairment and reducing the risk of complications, such as postoperative hemorrhage or dysphagia. Therefore, for patients prioritizing organ preservation and aesthetic outcomes, as illustrated by our case, induction chemotherapy followed by TORS represents an optimal treatment strategy.

In our case, fine-needle aspiration (FNA) of the thyroid gland revealed atypia, classified as Bethesda Category III (AUS) [7]. According to existing guidelines, this category implies a malignancy risk ranging from 13 to 30%, warranting repeat FNA, molecular testing, or diagnostic lobectomy as recommended follow-up strategies [7]. Following multidisciplinary consultation, including hematology expertise and shared decision-making with our patient, the management of the thyroid lesion was deferred until completion of definitive treatment for OPSCC. Subsequent histopathological analysis of a residual cervical lymph node demonstrated microscopic foci comprising well-formed thyroid follicles with colloid production, localized primarily in the subcapsular sinuses. The follicular cells lining these follicles exhibited mild atypia. A diagnosis of well-differentiated thyroid carcinoma of follicular cell origin, encompassing follicular variant papillary thyroid carcinoma (FV-PTC), and fusion-associated PTC was highly suspected. Given the presence of lateral cervical lymph node (level II and III) metastases and diagnostic uncertainty regarding thyroid pathology, bilateral total thyroidectomy was performed. Pathological examination confirmed the diagnosis as classic subtype PTC.

In our case, CT initially revealed an enlarged lymph node suspicious for OPSCC metastasis at level IIB, located posterior to the jugular vein. The lymph node metastases originating from tonsillar SCC predominantly involve ipsilateral level II nodes [12,13], followed sequentially by level III and IV nodes, for which ipsilateral selective neck dissection of levels II, III, and IV may suffice for N0 or N1 disease [12]. Comparatively, lymph node metastases from PTC are common, occurring in approximately 30% of patients, with micro metastases detectable in up to 80% [14,15]. Typically, in DTC, lymphatic metastasis initially involves level VI nodes ipsilaterally, subsequently spreading contralaterally to paratracheal lymph nodes [14, 16]. After level VI involvement, dissemination to level VII (subcarinal nodes) generally follows. Further metastatic spread commonly involves levels IV, III, IIA, and VB, with occasional metastasis to levels IIB and VA [14, 15]. Reported prevalence rates for lymph node metastases at levels IIA, IIB are approximately 53.1%, 15.5% respectively [15,17]. Some ectopic locations are also reported, which include retropharyngeal, parapharyngeal, retrocarotid, sublingual, axillary, and intracarotid locations [14]. In our case, the initially lymph node metastasis was IIB, which is not commonly seen in PTC.

Systemic therapy is not considered the first-line treatment for PTC. Chemotherapy is typically reserved for advanced cases unresponsive to both RAI and targeted therapies. Approximately 60% of patients with aggressive, metastatic DTC ultimately develop resistance to RAI therapy [18]. Such resistance may result from genetic mutations, impaired iodine transport into tumor cells, or interactions within the tumor microenvironment. Recent advancements in molecular biotechnology have facilitated novel therapeutic strategies to overcome radioactive iodine resistance. These approaches include redifferentiation therapies, targeted tyrosine kinase inhibitors (TKIs), and innovative drug delivery mechanisms, such as extracellular vesicle-mediated delivery systems [18]. Despite these advances, traditional cytotoxic chemotherapy remains largely ineffective for PTC and is generally not recommended as an initial treatment option [19].

Several studies report minimal responsiveness of DTC to cytotoxic chemotherapeutic agents [19]. This poor response is primarily attributed to the typically slow-growing nature of PTC, which contrasts with the rapid proliferative targets favored by cytotoxic agents, such as taxanes and platinum-based compounds. Our patient responded well to induction chemotherapy targeting the HPV-positive OPSCC, while the lymph node involvement likely arising from PTC exhibited only a partial response. One possible explanation for this atypical finding is tumor heterogeneity, where specific subpopulations of thyroid cancer cells may possess sensitivity to cytotoxic agents [20]. Further research is needed to better understand the molecular mechanisms that contribute to chemotherapeutic responsiveness in PTC.

## Conclusions

This report demonstrates a rare example of synchronous HPV-positive OPSCC and occult PTC. The initial presentation of cervical lymphadenopathy was presumed to originate solely from the oropharyngeal primary; however, subsequent pathological evaluation revealed that a subset of lymph node metastases originated from a clinically occult PTC. Careful pathological evaluation and multidisciplinary planning were essential for accurate diagnosis and treatment. The patient exhibited a favorable response to induction chemotherapy. This report underscores the importance of comprehensive evaluation of cervical lymphadenopathy and highlights the potential for multiple synchronous primary malignancies in head and neck oncology.
